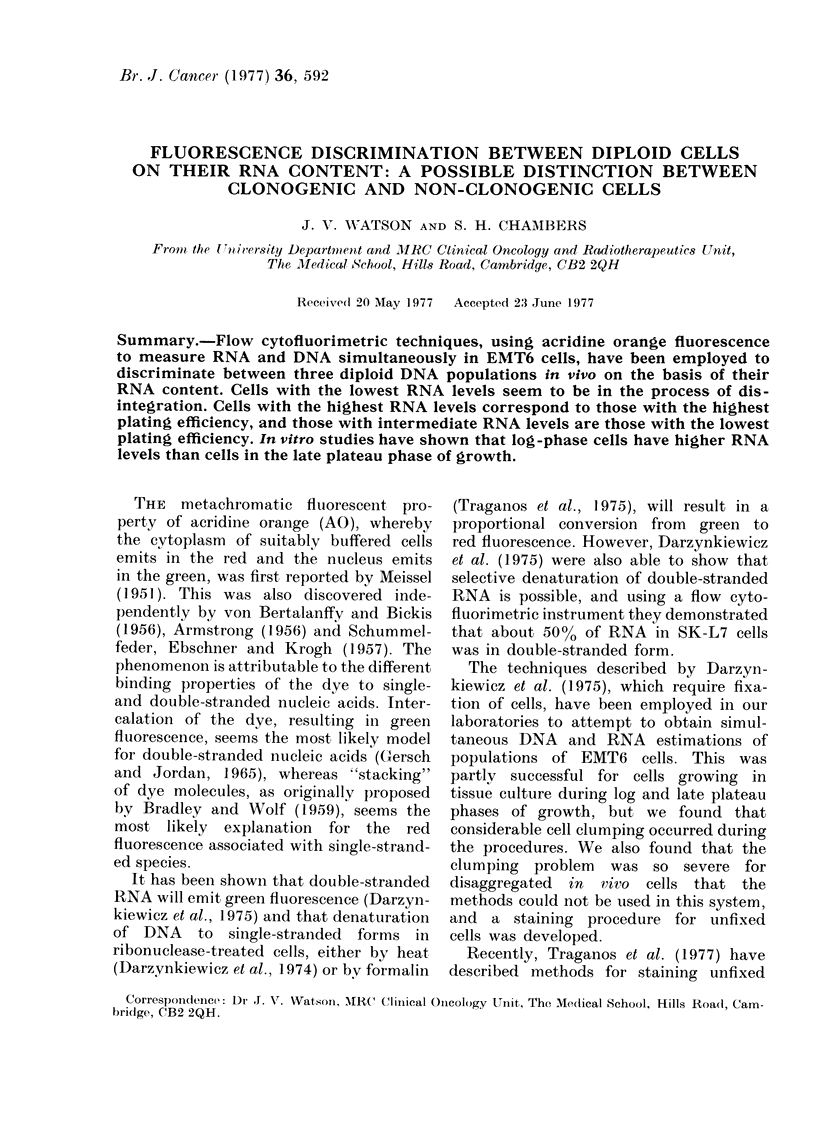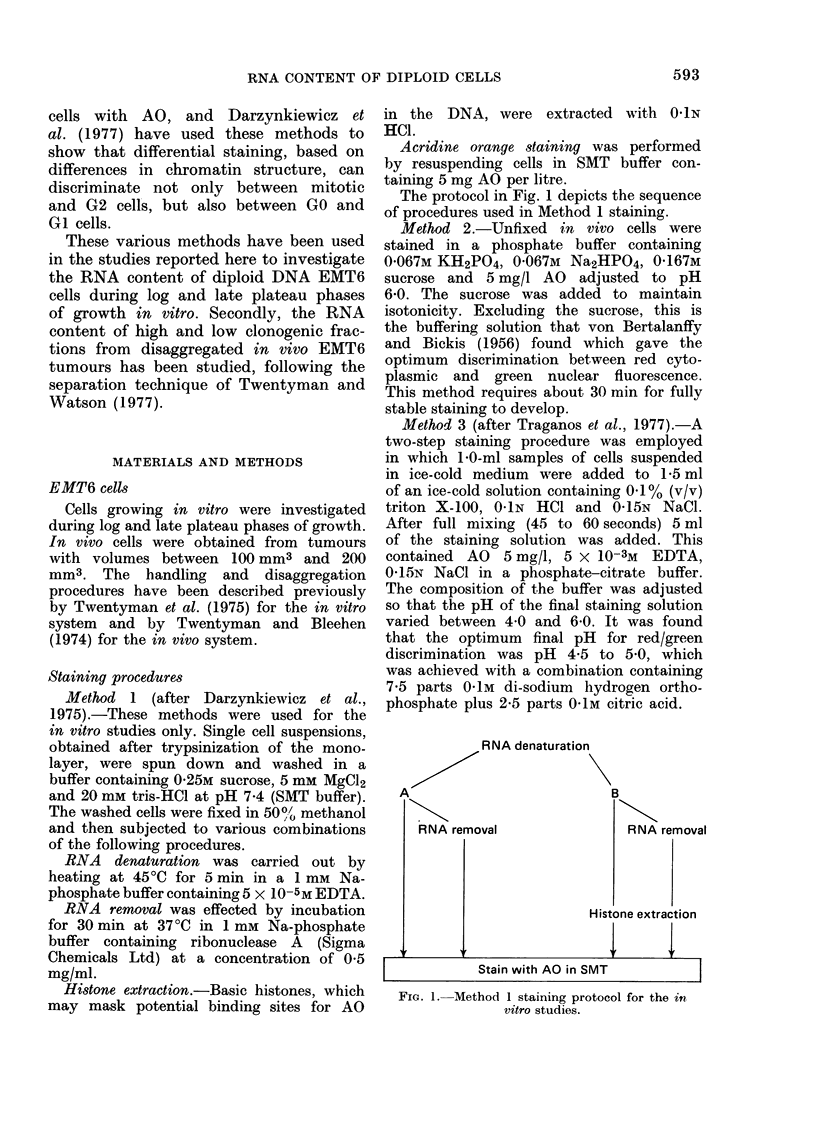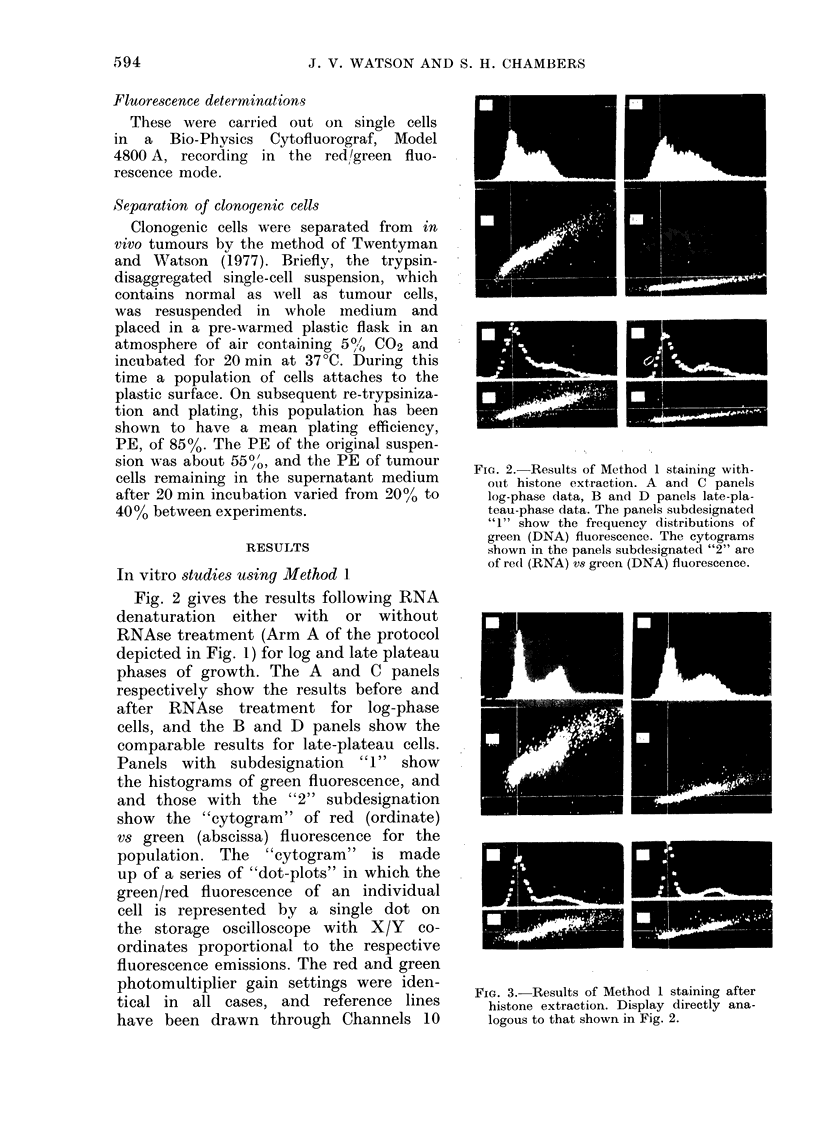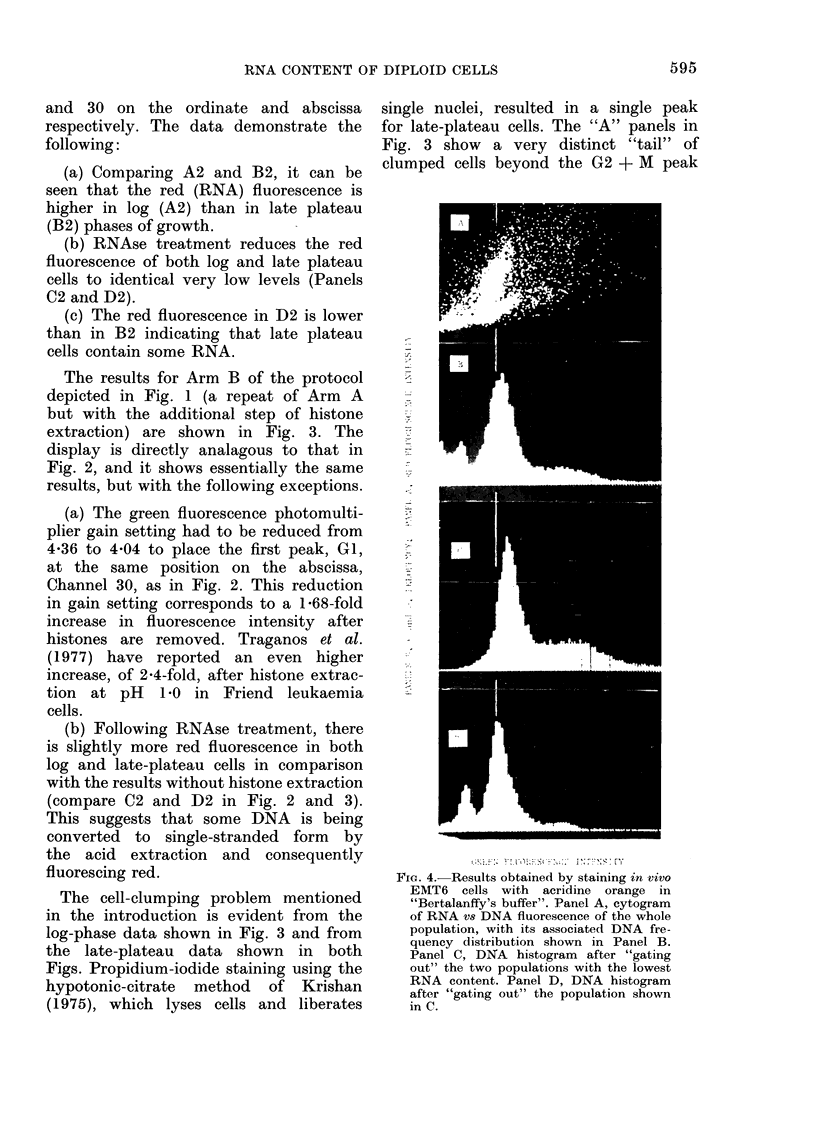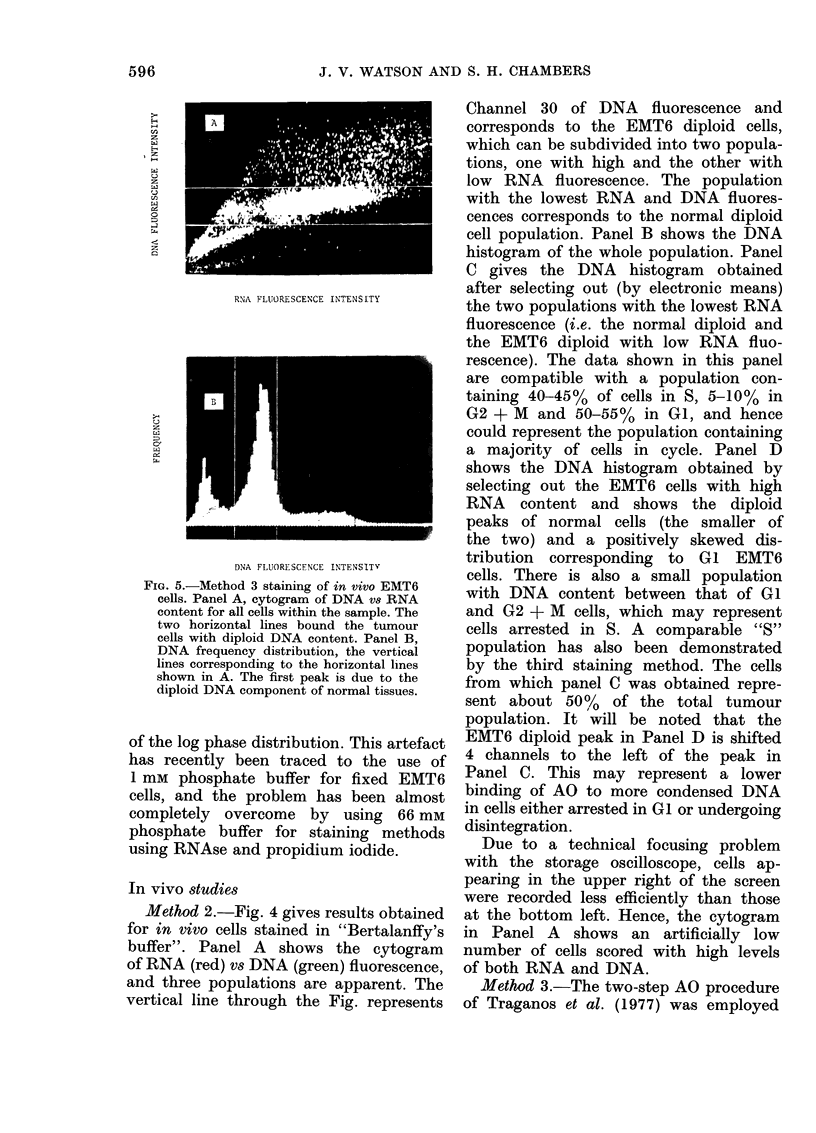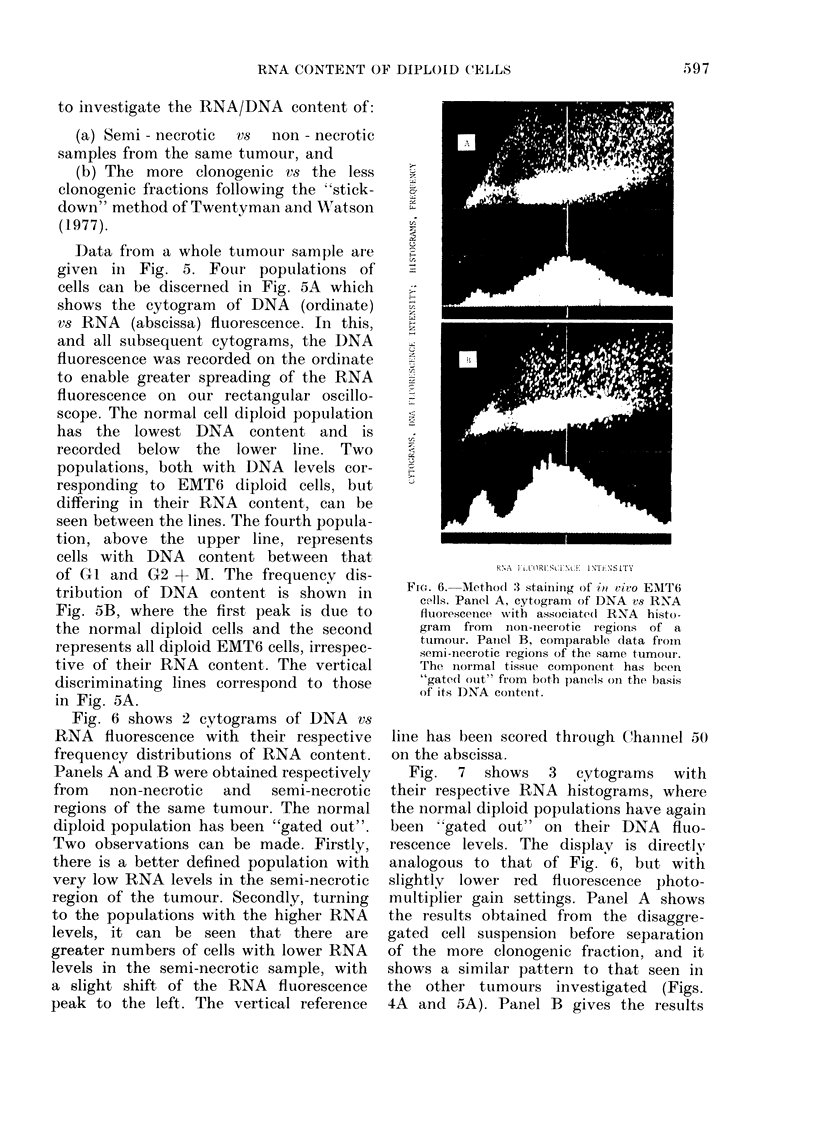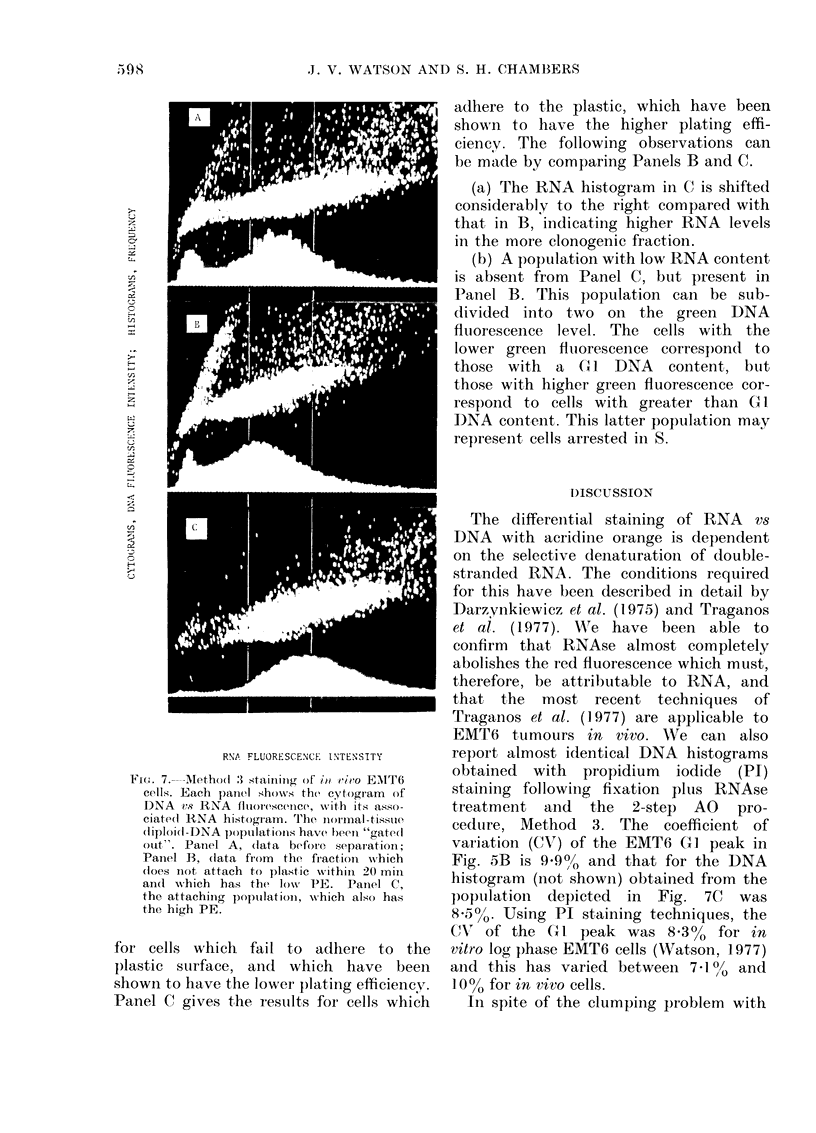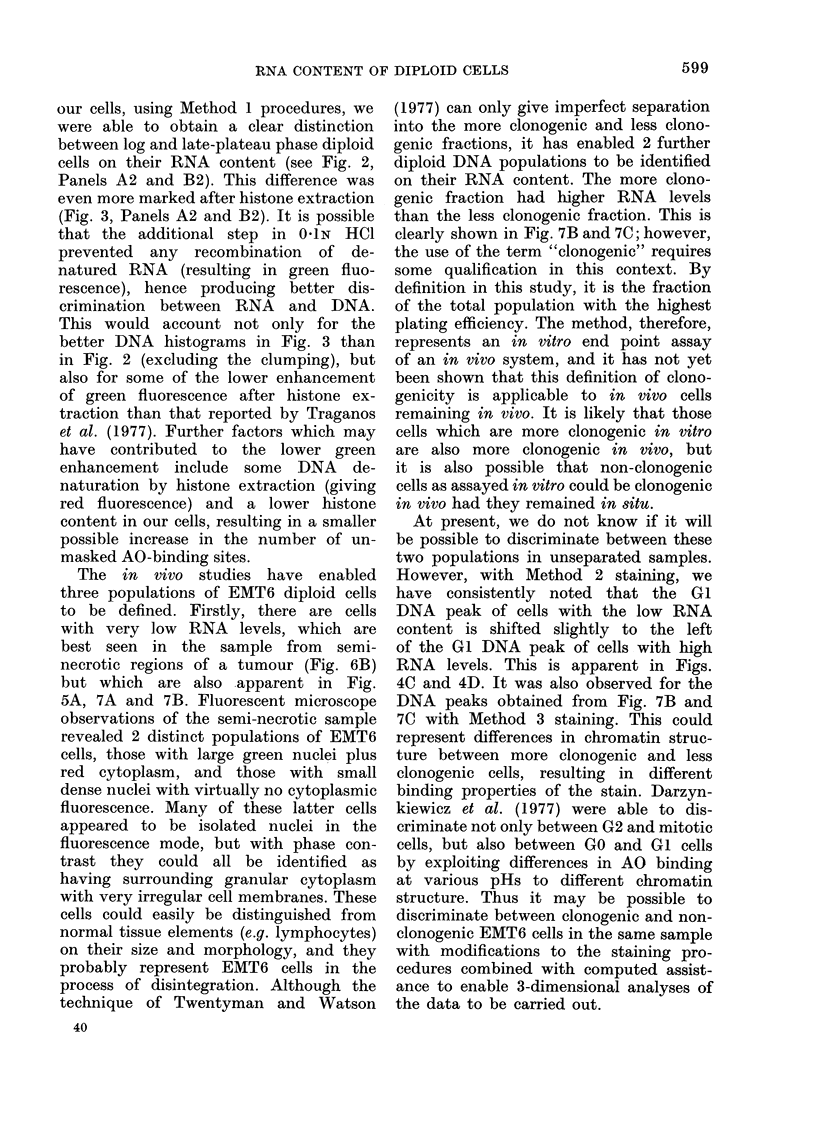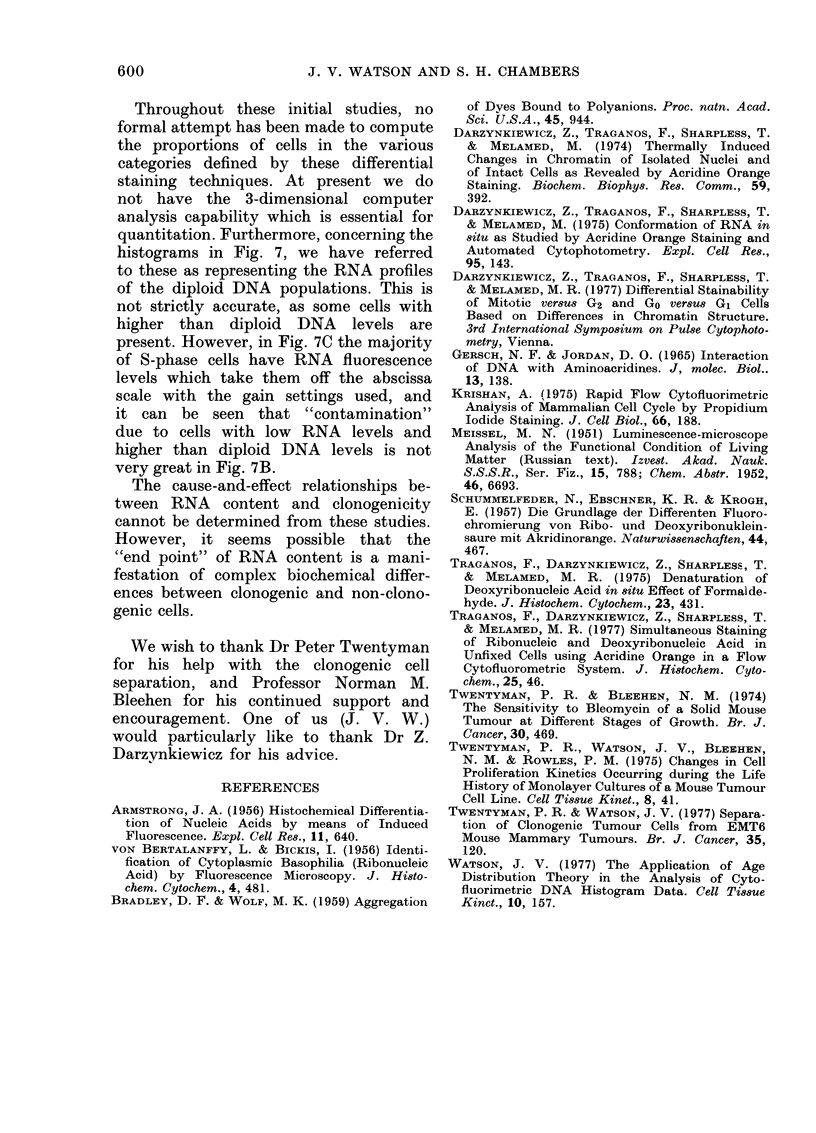# Fluorescence discrimination between diploid cells on their RNA content: a possible distinction between clonogenic and non-clonogenic cells.

**DOI:** 10.1038/bjc.1977.236

**Published:** 1977-11

**Authors:** J. V. Watson, S. H. Chambers

## Abstract

**Images:**


*Br. J. Cancer* (1977) **36**, 592

### FLUORESCENCE DISCRIMINATION BETWEEN DIPLOID CELLS ON THEIR RNA CONTENT: A POSSIBLE DISTINCTION BETWEEN CLONOGENIC AND NON-CLONOGENIC CELLS

J. V. WATSON AND S. H. CHAMBERS

*From the University Department and MRC Clinical Oncology and Radiotherapeutics Unit, The Medical School, Hills Road, Cambridge, CB2 2QH*

Received 20 May 1977 Accepted 23 June 1977

**Summary.—Flow cytofluorimetric techniques, using acridine orange fluorescence to measure RNA and DNA simultaneously in EMT6 cells, have been employed to discriminate between three diploid DNA populations *in vivo* on the basis of their RNA content. Cells with the lowest RNA levels seem to be in the process of disintegration. Cells with the highest RNA levels correspond to those with the highest plating efficiency, and those with intermediate RNA levels are those with the lowest plating efficiency. *In vitro* studies have shown that log-phase cells have higher RNA levels than cells in the late plateau phase of growth.**

THE metachromatic fluorescent property of acridine orange (AO), whereby the cytoplasm of suitably buffered cells emits in the red and the nucleus emits in the green, was first reported by Meissel (1951). This was also discovered independently by von Bertalanffy and Bickis (1956), Armstrong (1956) and Schummelfeder, Ebschner and Krogh (1957). The phenomenon is attributable to the different binding properties of the dye to single- and double-stranded nucleic acids. Intercalation of the dye, resulting in green fluorescence, seems the most likely model for double-stranded nucleic acids (Gersch and Jordan, 1965), whereas "stacking" of dye molecules, as originally proposed by Bradley and Wolf (1959), seems the most likely explanation for the red fluorescence associated with single-stranded species.

It has been shown that double-stranded RNA will emit green fluorescence (Darzynkiewicz *et al.*, 1975) and that denaturation of DNA to single-stranded forms in ribonuclease-treated cells, either by heat (Darzynkiewicz *et al.*, 1974) or by formalin (Traganos *et al.*, 1975), will result in a proportional conversion from green to red fluorescence. However, Darzynkiewicz *et al.* (1975) were also able to show that selective denaturation of double-stranded RNA is possible, and using a flow cytofluorimetric instrument they demonstrated that about 50% of RNA in SK-L7 cells was in double-stranded form.

The techniques described by Darzynkiewicz *et al.* (1975), which require fixation of cells, have been employed in our laboratories to attempt to obtain simultaneous DNA and RNA estimations of populations of EMT6 cells. This was partly successful for cells growing in tissue culture during log and late plateau phases of growth, but we found that considerable cell clumping occurred during the procedures. We also found that the clumping problem was so severe for disaggregated *in vivo* cells that the methods could not be used in this system, and a staining procedure for unfixed cells was developed.

Recently, Traganos *et al.* (1977) have described methods for staining unfixed

Correspondence: Dr J. V. Watson, MRC Clinical Oncology Unit, The Medical School, Hills Road, Cambridge, CB2 2QH.

cells with AO, and Darzynkiewicz *et al.* (1977) have used these methods to show that differential staining, based on differences in chromatin structure, can discriminate not only between mitotic and G2 cells, but also between G0 and G1 cells.

These various methods have been used in the studies reported here to investigate the RNA content of diploid DNA EMT6 cells during log and late plateau phases of growth *in vitro*. Secondly, the RNA content of high and low clonogenic fractions from disaggregated *in vivo* EMT6 tumours has been studied, following the separation technique of Twentyman and Watson (1977).

### MATERIALS AND METHODS

### *EMT6 cells*

Cells growing *in vitro* were investigated during log and late plateau phases of growth. *In vivo* cells were obtained from tumours with volumes between 100 $mm^3$ and 200 $mm^3$. The handling and disaggregation procedures have been described previously by Twentyman *et al.* (1975) for the *in vitro* system and by Twentyman and Bleehen (1974) for the *in vivo* system.

### *Staining procedures*

*Method* 1 (after Darzynkiewicz *et al.*, 1975).—These methods were used for the *in vitro* studies only. Single cell suspensions, obtained after trypsinization of the monolayer, were spun down and washed in a buffer containing 0·25M sucrose, 5 mM $MgCl_2$ and 20 mM tris-HCl at pH 7·4 (SMT buffer). The washed cells were fixed in 50% methanol and then subjected to various combinations of the following procedures.

*RNA denaturation* was carried out by heating at 45°C for 5 min in a 1 mM Na-phosphate buffer containing $5 \times 10^{-5}$M EDTA.

*RNA removal* was effected by incubation for 30 min at 37°C in 1 mM Na-phosphate buffer containing ribonuclease A (Sigma Chemicals Ltd) at a concentration of 0·5 mg/ml.

*Histone extraction.*—Basic histones, which may mask potential binding sites for AO in the DNA, were extracted with 0·1N HCl.

*Acridine orange staining* was performed by resuspending cells in SMT buffer containing 5 mg AO per litre.

The protocol in Fig. 1 depicts the sequence of procedures used in Method 1 staining.

*Method* 2.—Unfixed *in vivo* cells were stained in a phosphate buffer containing 0·067M $KH_2PO_4$, 0·067M $Na_2HPO_4$, 0·167M sucrose and 5 mg/l AO adjusted to pH 6·0. The sucrose was added to maintain isotonicity. Excluding the sucrose, this is the buffering solution that von Bertalanffy and Bickis (1956) found which gave the optimum discrimination between red cytoplasmic and green nuclear fluorescence. This method requires about 30 min for fully stable staining to develop.

*Method* 3 (after Traganos *et al.*, 1977).—A two-step staining procedure was employed in which 1·0-ml samples of cells suspended in ice-cold medium were added to 1·5 ml of an ice-cold solution containing 0·1% (v/v) triton X-100, 0·1N HCl and 0·15N NaCl. After full mixing (45 to 60 seconds) 5 ml of the staining solution was added. This contained AO 5 mg/l, $5 \times 10^{-3}$M EDTA, 0·15N NaCl in a phosphate–citrate buffer. The composition of the buffer was adjusted so that the pH of the final staining solution varied between 4·0 and 6·0. It was found that the optimum final pH for red/green discrimination was pH 4·5 to 5·0, which was achieved with a combination containing 7·5 parts 0·1M di-sodium hydrogen orthophosphate plus 2·5 parts 0·1M citric acid.

FIG. 1.—Method 1 staining protocol for the *in vitro* studies.

*Fluorescence determinations*

These were carried out on single cells in a Bio-Physics Cytofluorograf, Model 4800 A, recording in the red/green fluorescence mode.

*Separation of clonogenic cells*

Clonogenic cells were separated from *in vivo* tumours by the method of Twentyman and Watson (1977). Briefly, the trypsin-disaggregated single-cell suspension, which contains normal as well as tumour cells, was resuspended in whole medium and placed in a pre-warmed plastic flask in an atmosphere of air containing 5% $CO_2$ and incubated for 20 min at 37°C. During this time a population of cells attaches to the plastic surface. On subsequent re-trypsinization and plating, this population has been shown to have a mean plating efficiency, PE, of 85%. The PE of the original suspension was about 55%, and the PE of tumour cells remaining in the supernatant medium after 20 min incubation varied from 20% to 40% between experiments.

### RESULTS

### In vitro *studies using Method 1*

Fig. 2 gives the results following RNA denaturation either with or without RNAse treatment (Arm A of the protocol depicted in Fig. 1) for log and late plateau phases of growth. The A and C panels respectively show the results before and after RNAse treatment for log-phase cells, and the B and D panels show the comparable results for late-plateau cells. Panels with subdesignation "1" show the histograms of green fluorescence, and and those with the "2" subdesignation show the "cytogram" of red (ordinate) *vs* green (abscissa) fluorescence for the population. The "cytogram" is made up of a series of "dot-plots" in which the green/red fluorescence of an individual cell is represented by a single dot on the storage oscilloscope with X/Y coordinates proportional to the respective fluorescence emissions. The red and green photomultiplier gain settings were identical in all cases, and reference lines have been drawn through Channels 10

FIG. 2.—Results of Method 1 staining without histone extraction. A and C panels log-phase data, B and D panels late-plateau-phase data. The panels subdesignated "1" show the frequency distributions of green (DNA) fluorescence. The cytograms shown in the panels subdesignated "2" are of red (RNA) *vs* green (DNA) fluorescence.

FIG. 3.—Results of Method 1 staining after histone extraction. Display directly analogous to that shown in Fig. 2.

and 30 on the ordinate and abscissa respectively. The data demonstrate the following:

(a) Comparing A2 and B2, it can be seen that the red (RNA) fluorescence is higher in log (A2) than in late plateau (B2) phases of growth.

(b) RNAse treatment reduces the red fluorescence of both log and late plateau cells to identical very low levels (Panels C2 and D2).

(c) The red fluorescence in D2 is lower than in B2 indicating that late plateau cells contain some RNA.

The results for Arm B of the protocol depicted in Fig. 1 (a repeat of Arm A but with the additional step of histone extraction) are shown in Fig. 3. The display is directly analagous to that in Fig. 2, and it shows essentially the same results, but with the following exceptions.

(a) The green fluorescence photomultiplier gain setting had to be reduced from 4·36 to 4·04 to place the first peak, G1, at the same position on the abscissa, Channel 30, as in Fig. 2. This reduction in gain setting corresponds to a 1·68-fold increase in fluorescence intensity after histones are removed. Traganos *et al.* (1977) have reported an even higher increase, of 2·4-fold, after histone extraction at pH 1·0 in Friend leukaemia cells.

(b) Following RNAse treatment, there is slightly more red fluorescence in both log and late-plateau cells in comparison with the results without histone extraction (compare C2 and D2 in Fig. 2 and 3). This suggests that some DNA is being converted to single-stranded form by the acid extraction and consequently fluorescing red.

The cell-clumping problem mentioned in the introduction is evident from the log-phase data shown in Fig. 3 and from the late-plateau data shown in both Figs. Propidium-iodide staining using the hypotonic-citrate method of Krishan (1975), which lyses cells and liberates single nuclei, resulted in a single peak for late-plateau cells. The "A" panels in Fig. 3 show a very distinct "tail" of clumped cells beyond the G2 + M peak

FIG. 4.—Results obtained by staining *in vivo* EMT6 cells with acridine orange in "Bertalanffy's buffer". Panel A, cytogram of RNA *vs* DNA fluorescence of the whole population, with its associated DNA frequency distribution shown in Panel B. Panel C, DNA histogram after "gating out" the two populations with the lowest RNA content. Panel D, DNA histogram after "gating out" the population shown in C.

FIG. 5.—Method 3 staining of *in vivo* EMT6 cells. Panel A, cytogram of DNA *vs* RNA content for all cells within the sample. The two horizontal lines bound the tumour cells with diploid DNA content. Panel B, DNA frequency distribution, the vertical lines corresponding to the horizontal lines shown in A. The first peak is due to the diploid DNA component of normal tissues.

of the log phase distribution. This artefact has recently been traced to the use of 1 mM phosphate buffer for fixed EMT6 cells, and the problem has been almost completely overcome by using 66 mM phosphate buffer for staining methods using RNAse and propidium iodide.

### In vivo *studies*

*Method* 2.—Fig. 4 gives results obtained for *in vivo* cells stained in "Bertalanffy's buffer". Panel A shows the cytogram of RNA (red) *vs* DNA (green) fluorescence, and three populations are apparent. The vertical line through the Fig. represents Channel 30 of DNA fluorescence and corresponds to the EMT6 diploid cells, which can be subdivided into two populations, one with high and the other with low RNA fluorescence. The population with the lowest RNA and DNA fluorescences corresponds to the normal diploid cell population. Panel B shows the DNA histogram of the whole population. Panel C gives the DNA histogram obtained after selecting out (by electronic means) the two populations with the lowest RNA fluorescence (*i.e.* the normal diploid and the EMT6 diploid with low RNA fluorescence). The data shown in this panel are compatible with a population containing 40–45% of cells in S, 5–10% in G2 + M and 50–55% in G1, and hence could represent the population containing a majority of cells in cycle. Panel D shows the DNA histogram obtained by selecting out the EMT6 cells with high RNA content and shows the diploid peaks of normal cells (the smaller of the two) and a positively skewed distribution corresponding to G1 EMT6 cells. There is also a small population with DNA content between that of G1 and G2 + M cells, which may represent cells arrested in S. A comparable "S" population has also been demonstrated by the third staining method. The cells from which panel C was obtained represent about 50% of the total tumour population. It will be noted that the EMT6 diploid peak in Panel D is shifted 4 channels to the left of the peak in Panel C. This may represent a lower binding of AO to more condensed DNA in cells either arrested in G1 or undergoing disintegration.

Due to a technical focusing problem with the storage oscilloscope, cells appearing in the upper right of the screen were recorded less efficiently than those at the bottom left. Hence, the cytogram in Panel A shows an artificially low number of cells scored with high levels of both RNA and DNA.

*Method* 3.—The two-step AO procedure of Traganos *et al.* (1977) was employed to investigate the RNA/DNA content of:

(a) Semi - necrotic *vs* non - necrotic samples from the same tumour, and

(b) The more clonogenic *vs* the less clonogenic fractions following the "stick-down" method of Twentyman and Watson (1977).

Data from a whole tumour sample are given in Fig. 5. Four populations of cells can be discerned in Fig. 5A which shows the cytogram of DNA (ordinate) *vs* RNA (abscissa) fluorescence. In this, and all subsequent cytograms, the DNA fluorescence was recorded on the ordinate to enable greater spreading of the RNA fluorescence on our rectangular oscilloscope. The normal cell diploid population has the lowest DNA content and is recorded below the lower line. Two populations, both with DNA levels corresponding to EMT6 diploid cells, but differing in their RNA content, can be seen between the lines. The fourth population, above the upper line, represents cells with DNA content between that of G1 and G2 + M. The frequency distribution of DNA content is shown in Fig. 5B, where the first peak is due to the normal diploid cells and the second represents all diploid EMT6 cells, irrespective of their RNA content. The vertical discriminating lines correspond to those in Fig. 5A.

Fig. 6 shows 2 cytograms of DNA *vs* RNA fluorescence with their respective frequency distributions of RNA content. Panels A and B were obtained respectively from non-necrotic and semi-necrotic regions of the same tumour. The normal diploid population has been "gated out". Two observations can be made. Firstly, there is a better defined population with very low RNA levels in the semi-necrotic region of the tumour. Secondly, turning to the populations with the higher RNA levels, it can be seen that there are greater numbers of cells with lower RNA levels in the semi-necrotic sample, with a slight shift of the RNA fluorescence peak to the left. The vertical reference

FIG. 6.—Method 3 staining of *in vivo* EMT6 cells. Panel A, cytogram of DNA *vs* RNA fluorescence with associated RNA histogram from non-necrotic regions of a tumour. Panel B, comparable data from semi-necrotic regions of the same tumour. The normal tissue component has been "gated out" from both panels on the basis of its DNA content.

line has been scored through Channel 50 on the abscissa.

Fig. 7 shows 3 cytograms with their respective RNA histograms, where the normal diploid populations have again been "gated out" on their DNA fluorescence levels. The display is directly analogous to that of Fig. 6, but with slightly lower red fluorescence photomultiplier gain settings. Panel A shows the results obtained from the disaggregated cell suspension before separation of the more clonogenic fraction, and it shows a similar pattern to that seen in the other tumours investigated (Figs. 4A and 5A). Panel B gives the results

FIG. 7.—Method 3 staining of *in vivo* EMT6 cells. Each panel shows the cytogram of DNA *vs* RNA fluorescence, with its associated RNA histogram. The normal-tissue diploid-DNA populations have been "gated out". Panel A, data before separation; Panel B, data from the fraction which does not attach to plastic within 20 min and which has the low PE. Panel C, the attaching population, which also has the high PE.

for cells which fail to adhere to the plastic surface, and which have been shown to have the lower plating efficiency. Panel C gives the results for cells which adhere to the plastic, which have been shown to have the higher plating efficiency. The following observations can be made by comparing Panels B and C.

(a) The RNA histogram in C is shifted considerably to the right compared with that in B, indicating higher RNA levels in the more clonogenic fraction.

(b) A population with low RNA content is absent from Panel C, but present in Panel B. This population can be subdivided into two on the green DNA fluorescence level. The cells with the lower green fluorescence correspond to those with a G1 DNA content, but those with higher green fluorescence correspond to cells with greater than G1 DNA content. This latter population may represent cells arrested in S.

### DISCUSSION

The differential staining of RNA *vs* DNA with acridine orange is dependent on the selective denaturation of double-stranded RNA. The conditions required for this have been described in detail by Darzynkiewicz *et al.* (1975) and Traganos *et al.* (1977). We have been able to confirm that RNAse almost completely abolishes the red fluorescence which must, therefore, be attributable to RNA, and that the most recent techniques of Traganos *et al.* (1977) are applicable to EMT6 tumours *in vivo*. We can also report almost identical DNA histograms obtained with propidium iodide (PI) staining following fixation plus RNAse treatment and the 2-step AO procedure, Method 3. The coefficient of variation (CV) of the EMT6 G1 peak in Fig. 5B is 9·9% and that for the DNA histogram (not shown) obtained from the population depicted in Fig. 7C was 8·5%. Using PI staining techniques, the CV of the G1 peak was 8·3% for *in vitro* log phase EMT6 cells (Watson, 1977) and this has varied between 7·1% and 10% for *in vivo* cells.

In spite of the clumping problem with our cells, using Method 1 procedures, we were able to obtain a clear distinction between log and late-plateau phase diploid cells on their RNA content (see Fig. 2, Panels A2 and B2). This difference was even more marked after histone extraction (Fig. 3, Panels A2 and B2). It is possible that the additional step in 0·1N HCl prevented any recombination of denatured RNA (resulting in green fluorescence), hence producing better discrimination between RNA and DNA. This would account not only for the better DNA histograms in Fig. 3 than in Fig. 2 (excluding the clumping), but also for some of the lower enhancement of green fluorescence after histone extraction than that reported by Traganos *et al.* (1977). Further factors which may have contributed to the lower green enhancement include some DNA denaturation by histone extraction (giving red fluorescence) and a lower histone content in our cells, resulting in a smaller possible increase in the number of unmasked AO-binding sites.

The *in vivo* studies have enabled three populations of EMT6 diploid cells to be defined. Firstly, there are cells with very low RNA levels, which are best seen in the sample from semi-necrotic regions of a tumour (Fig. 6B) but which are also apparent in Fig. 5A, 7A and 7B. Fluorescent microscope observations of the semi-necrotic sample revealed 2 distinct populations of EMT6 cells, those with large green nuclei plus red cytoplasm, and those with small dense nuclei with virtually no cytoplasmic fluorescence. Many of these latter cells appeared to be isolated nuclei in the fluorescence mode, but with phase contrast they could all be identified as having surrounding granular cytoplasm with very irregular cell membranes. These cells could easily be distinguished from normal tissue elements (*e.g.* lymphocytes) on their size and morphology, and they probably represent EMT6 cells in the process of disintegration. Although the technique of Twentyman and Watson (1977) can only give imperfect separation into the more clonogenic and less clonogenic fractions, it has enabled 2 further diploid DNA populations to be identified on their RNA content. The more clonogenic fraction had higher RNA levels than the less clonogenic fraction. This is clearly shown in Fig. 7B and 7C; however, the use of the term "clonogenic" requires some qualification in this context. By definition in this study, it is the fraction of the total population with the highest plating efficiency. The method, therefore, represents an *in vitro* end point assay of an *in vivo* system, and it has not yet been shown that this definition of clonogenicity is applicable to *in vivo* cells remaining *in vivo*. It is likely that those cells which are more clonogenic *in vitro* are also more clonogenic *in vivo*, but it is also possible that non-clonogenic cells as assayed *in vitro* could be clonogenic *in vivo* had they remained *in situ*.

At present, we do not know if it will be possible to discriminate between these two populations in unseparated samples. However, with Method 2 staining, we have consistently noted that the G1 DNA peak of cells with the low RNA content is shifted slightly to the left of the G1 DNA peak of cells with high RNA levels. This is apparent in Figs. 4C and 4D. It was also observed for the DNA peaks obtained from Fig. 7B and 7C with Method 3 staining. This could represent differences in chromatin structure between more clonogenic and less clonogenic cells, resulting in different binding properties of the stain. Darzynkiewicz *et al.* (1977) were able to discriminate not only between G2 and mitotic cells, but also between G0 and G1 cells by exploiting differences in AO binding at various pHs to different chromatin structure. Thus it may be possible to discriminate between clonogenic and non-clonogenic EMT6 cells in the same sample with modifications to the staining procedures combined with computed assistance to enable 3-dimensional analyses of the data to be carried out.

Throughout these initial studies, no formal attempt has been made to compute the proportions of cells in the various categories defined by these differential staining techniques. At present we do not have the 3-dimensional computer analysis capability which is essential for quantitation. Furthermore, concerning the histograms in Fig. 7, we have referred to these as representing the RNA profiles of the diploid DNA populations. This is not strictly accurate, as some cells with higher than diploid DNA levels are present. However, in Fig. 7C the majority of S-phase cells have RNA fluorescence levels which take them off the abscissa scale with the gain settings used, and it can be seen that "contamination" due to cells with low RNA levels and higher than diploid DNA levels is not very great in Fig. 7B.

The cause-and-effect relationships between RNA content and clonogenicity cannot be determined from these studies. However, it seems possible that the "end point" of RNA content is a manifestation of complex biochemical differences between clonogenic and non-clonogenic cells.

We wish to thank Dr Peter Twentyman for his help with the clonogenic cell separation, and Professor Norman M. Bleehen for his continued support and encouragement. One of us (J. V. W.) would particularly like to thank Dr Z. Darzynkiewicz for his advice.

REFERENCES

ARMSTRONG, J. A. (1956) Histochemical Differentiation of Nucleic Acids by means of Induced Fluorescence. *Expl. Cell Res.*, **11**, 640.

VON BERTALANFFY, L. & BICKIS, I. (1956) Identification of Cytoplasmic Basophilia (Ribonucleic Acid) by Fluorescence Microscopy. *J. Histochem. Cytochem.*, **4**, 481.

BRADLEY, D. F. & WOLF, M. K. (1959) Aggregation of Dyes Bound to Polyanions. *Proc. natn. Acad. Sci. U.S.A.*, **45**, 944.

DARZYNKIEWICZ, Z., TRAGANOS, F., SHARPLESS, T. & MELAMED, M. (1974) Thermally Induced Changes in Chromatin of Isolated Nuclei and of Intact Cells as Revealed by Acridine Orange Staining. *Biochem. Biophys. Res. Comm.*, **59**, 392.

DARZYNKIEWICZ, Z., TRAGANOS, F., SHARPLESS, T. & MELAMED, M. (1975) Conformation of RNA *in situ* as Studied by Acridine Orange Staining and Automated Cytophotometry. *Expl. Cell Res.*, **95**, 143.

DARZYNKIEWICZ, Z., TRAGANOS, F., SHARPLESS, T. & MELAMED, M. R. (1977) Differential Stainability of Mitotic *versus* $G_2$ and $G_0$ *versus* $G_1$ Cells Based on Differences in Chromatin Structure. *3rd International Symposium on Pulse Cytophotometry*, Vienna.

GERSCH, N. F. & JORDAN, D. O. (1965) Interaction of DNA with Aminoacridines. *J, molec. Biol..* **13**, 138.

KRISHAN, A. (1975) Rapid Flow Cytofluorimetric Analysis of Mammalian Cell Cycle by Propidium Iodide Staining. *J. Cell Biol.*, **66**, 188.

MEISSEL, M. N. (1951) Luminescence-microscope Analysis of the Functional Condition of Living Matter (Russian text). *Izvest. Akad. Nauk. S.S.S.R.*, Ser. Fiz., **15**, 788; *Chem. Abstr.* 1952, **46**, 6693.

SCHUMMELFEDER, N., EBSCHNER, K. R. & KROGH, E. (1957) Die Grundlage der Differenten Fluorochromierung von Ribo- und Deoxyribonukleinsaure mit Akridinorange. *Naturwissenschaften*, **44**, 467.

TRAGANOS, F., DARZYNKIEWICZ, Z., SHARPLESS, T. & MELAMED, M. R. (1975) Denaturation of Deoxyribonucleic Acid *in situ* Effect of Formaldehyde. *J. Histochem. Cytochem.*, **23**, 431.

TRAGANOS, F., DARZYNKIEWICZ, Z., SHARPLESS, T. & MELAMED, M. R. (1977) Simultaneous Staining of Ribonucleic and Deoxyribonucleic Acid in Unfixed Cells using Acridine Orange in a Flow Cytofluorometric System. *J. Histochem. Cytochem.*, **25**, 46.

TWENTYMAN, P. R. & BLEEHEN, N. M. (1974) The Sensitivity to Bleomycin of a Solid Mouse Tumour at Different Stages of Growth. *Br. J. Cancer*, **30**, 469.

TWENTYMAN, P. R., WATSON, J. V., BLEEHEN, N. M. & ROWLES, P. M. (1975) Changes in Cell Proliferation Kinetics Occurring during the Life History of Monolayer Cultures of a Mouse Tumour Cell Line. *Cell Tissue Kinet.*, **8**, 41.

TWENTYMAN, P. R. & WATSON, J. V. (1977) Separation of Clonogenic Tumour Cells from EMT6 Mouse Mammary Tumours. *Br. J. Cancer*, **35**, 120.

WATSON, J. V. (1977) The Application of Age Distribution Theory in the Analysis of Cytofluorimetric DNA Histogram Data. *Cell Tissue Kinet.*, **10**, 157.